# Complete Genome Sequence of a Recombinant Porcine Epidemic Diarrhea Virus Strain, CH/JXJA/2017, Isolated in Jiangxi, China, in 2017

**DOI:** 10.1128/genomeA.01590-17

**Published:** 2018-02-08

**Authors:** Kai Li, Deping Song, Fanfan Zhang, Wang Gong, Nannan Guo, Anqi Li, Xingrong Zhou, Dongyan Huang, Yu Ye, Yuxin Tang

**Affiliations:** a Key Laboratory for Animal Health of Jiangxi Province, Nanchang, Jiangxi, China; b Department of Preventive Veterinary Medicine, College of Animal Science and Technology, Jiangxi Agricultural University, Nanchang, Jiangxi, China

## Abstract

The full-length genome sequence of a variant of porcine epidemic diarrhea virus (PEDV), that of strain CH/JXJA/2017, was highly homologous to CH/ZMDZY/11, a highly virulent Chinese PEDV strain. CH/JXJA/2017 had a distant relationship with the attenuated CV777 vaccine strain, but the insertion sites of the S1 gene were similar to those of the recombinant strain of CH/ZMDZY/11.

## GENOME ANNOUNCEMENT

Porcine epidemic diarrhea (PED) is a devastating enteric disease (1) caused by PED virus (PEDV), an RNA virus in the genus *Alphacoronavirus*, family *Coronaviridae* (2). Presently, PEDV is posing a threat to the swine industry worldwide (3–7). PED was first reported in England in 1971 (8) and has since been identified in many pig-raising countries, including European, American, and Asian countries (9). In 2010, the large-scale outbreaks of PED with high morbidity and mortality occurred in swine farms in China, resulting in huge economic losses (10, 11).

In April 2017, a PEDV outbreak occurred in a farrow-to-finish herd in southern China. The mortality rate of the disease ranged from 10% in weaning pigs to 95% in neonatal piglets. PEDV was identified by an established reverse transcription-PCR with primers specific to the N gene of PEDV. Subsequently, the whole-genome sequence of one representative strain of PEDV, designated CH/JXJA/2017, was determined according to the previously reported methodology (12), and the data were assembled and annotated by Lasergene version 7.10 (DNAStar, Inc., USA).

The complete genome sequence of CH/JXJA/2017 was 28,079 nucleotides (nt) in length, including the poly(A) tail. The genomic organization of the virus was in the following order: 5′-untranslated region (UTR) (nt 1 to 292), replicase polyprotein (nt 293 to 12601 for 1a and nt 12601 to 20637 for 1b), spike (S) protein (nt 20634 to 24794), open reading frame 3 (ORF3) (nt 24794 to 25468), envelope (E) protein (nt 25449 to 25679), membrane (M) protein (nt 25687 to 26367), nucleocapsid (N) (nt 26379 to 27704), 3′-UTR (nt 27705 to 28038), and a poly(A) tail (nt 28038 to 28079). To elucidate the genetic characterization of CH/JXJA/2017 and the evolutionary relationships with other PEDV strains, including classical vaccine strains and other field strains retrieved from GenBank, a multialignment analysis was performed by using Molecular Evolutionary Genetics Analysis 7 (MEGA 7) (13). The results indicated that the complete genome sequence of CH/JXJA/2017 shared the highest nucleotide identity (99.2%) with CH/ZMDZY/11 (GenBank accession number KC196276), a recombinant indel-like strain from China (11), and it had a nucleotide identity of 96.7% with the attenuated CV777 strain. Phylogenetic analysis based on the full-length genome sequences of PEDVs revealed that CH/JXJA/2017 belonged to genogroup 2 (G2). Interestingly, the CH/JXJA/2017 strain possessed two unique deletion and two insertion sites compared to the PEDV attenuated vaccine CV777 strain (nt 20811 to 20822, nt 21123 to 21128, nt 21054 to 21056, and nt 21105 to 21107) in the S gene, and one 4-amino-acid deletion (^59^QGVN^62^), one 2-amino-acid deletion (^163^NI^164^), and two single-amino-acid insertions (^140^N and ^157^H) were observed. Of note, the 4-amino-acid insertion site (^59^QGVN^62^) of CH/JXJA/2017 was in the S1 domain of the S protein, which was similar to CH/ZMDZY/11. These findings suggest that CH/JXJA/2017 was a novel PEDV variant. Further study is required to determine the biological properties of this novel variant of PEDV.

### Accession number(s).

The complete genome sequence of PEDV strain CH/JXJA/2017 has been deposited in GenBank under the accession number MF375374.
